# Draft Whole-Genome Sequence of *Deinococcus* sp. UR1, a Putative Novel Species Isolated from an External Stainless Steel Surface in the Canadian Prairies

**DOI:** 10.1128/genomeA.01407-17

**Published:** 2018-01-11

**Authors:** Danae M. Suchan, Joel J. Steve, Alexa J. Pierce, Stephen C. Olshefsky, Morgan W. B. Kirzinger, Benjamin J. Perry, Andrew D. S. Cameron, Christopher K. Yost

**Affiliations:** a Department of Biology, Institute for Microbial Systems and Society, University of Regina, Regina, Saskatchewan, Canada

## Abstract

*Deinococcus* sp. strain UR1, a resilient bacterium isolated from the surface of a stainless steel sign located on the University of Regina campus in Saskatchewan, Canada, was sequenced to 56-fold coverage to produce 73 contigs with a consensus length of 4,472,838 bp and a G+C content of 69.37%.

## GENOME ANNOUNCEMENT

*Deinococcus* represents a genus of ubiquitous bacteria that are resistant to extreme environmental stresses, such as radiation and desiccation. This tolerance is thought to be partly facilitated by proficient DNA repair mechanisms (1, 2). Herein, we report the whole-genome sequencing of *Deinococcus* sp. strain UR1, an environmental strain isolated during surface sampling of a stainless steel sign at the University of Regina (GPS coordinates, 50.415981, −104.594733). The strain was isolated during an undergraduate research-based environmental microbiology course. *Deinococcus* sp. UR1 can be cultured on both lysogeny broth and tryptone yeast agar media at 30°C and produces circular orange colonies composed of coccoid cells. Cells can be connected in chain-like filaments and stain as Gram variable. *Deinococcus* sp. UR1 is capable of growth following UVB radiation at 302 nm at a cumulative dose of 450 kJ/m^2^. Desiccation on a vacuum filter that was stored for 5 weeks under ambient conditions resulted in only a 2.8-log reduction in cell number. Following 16S rRNA classification as the genus *Deinococcus*, an average nucleotide identity using BLAST+ (ANIb) analysis of paired *Deinococcus* genomes was performed using JSpeciesWS (3) to obtain a species identification. The resulting *Deinococcus* sp. UR1 ANIb values were all below the 95% sequence identity species threshold. *Deinococcus* sp. UR1 has the highest degrees of genomic relatedness to *Deinococcus soli* and *Deinococcus actinosclerus*, with respective ANIb values of 82.77% and 82.55%.

Genomic DNA was obtained from a culture grown overnight in lysogeny broth using phenol-chloroform extraction and fragmented using a Bioruptor standard sonication device (Diagenode). DNA was prepared for sequencing using the NEBNext Ultra II DNA library prep kit (New England BioLabs) with a final library size selection of 600 bp, using Agencourt AMPure XP magnetic beads (Beckman Coulter, Inc.). Sequencing was performed with an Illumina MiSeq system and V3 300-bp paired-end sequencing chemistry. Trimmomatic version 0.35 (4) was used to quality trim the raw reads, removing adapter sequences and low-quality bases (*Q* score, <5) from the ends of reads and trimming at an average *Q* score of <15 in a 3-bp sliding window. This yielded a total of 861,950 high-quality paired reads for genome assembly. Filtered paired-end reads were assembled *de novo* using “careful” and “cov-cutoff” modes in SPAdes version 3.8.0 (5) with a *k*-mer size of 127 bp, which provided an average genome coverage of 56×. The resulting genome assembly produced 73 contigs with a consensus length of 4,472,838 bp (69.37% G+C content), an *N*_50_ value of 143,250 bp, and an *L*_75_ value of 20 contigs.

A draft genome sequence annotation was performed using the NCBI Prokaryotic Genome Annotation Pipeline (6), which predicted 3,982 protein-coding genes, 60 RNA-coding genes, and 106 pseudogenes. The draft genome sequence was also analyzed for prophage regions with the PHAge Search Tool Enhanced Release (PHASTER) server (7). PHASTER identified three incomplete prophage regions which shared similarity with recently published *Gordonia* phage sequences (8, 9) and *Salmonella* phage sequence 118970_sal2 (GenBank accession number NC_031933).

### Accession number(s).

This whole-genome shotgun project has been deposited in GenBank under the accession number LIYN00000000 and under the NCBI BioProject accession number PRJNA293368. The version described in this paper is version LIYN02000000.
